# Herbal formula Huangqi Guizhi Wuwu decoction attenuates paclitaxel-related neurotoxicity via inhibition of inflammation and oxidative stress

**DOI:** 10.1186/s13020-021-00488-1

**Published:** 2021-08-10

**Authors:** Zhangming Lv, Jiayun Shen, Xuejiao Gao, Yonglan Ruan, Jinying Ling, Rongwei Sun, Jingya Dai, Haizhen Fan, Xiaolan Cheng, Peng Cao

**Affiliations:** 1 Affiliated Hospital of Integrated Traditional Chinese and Western Medicine, Nanjing University of Chinese Medicine, Nanjing, 210028 China; 2 College of Pharmacy, Nanjing University of Chinese Medicine, Nanjing, 210029 China; 3 School of Chinese Medicine & School of Integrated Chinese and Western Medicine, Nanjing University of Chinese Medicine, Nanjing, 210023 China

**Keywords:** Huangqi Guizhi Wuwu decoction, Paclitaxel, Peripheral neuropathy, Network pharmacology, Inflammation, Oxidative stress

## Abstract

**Background:**

Paclitaxel-induced peripheral neuropathy (PIPN) is a challenging clinical problem during chemotherapy. Our previous work found that herbal formula Huangqi Guizhi Wuwu decoction (HGWD) could reduce oxaliplatin-induced neurotoxicity. However, its effect on PIPN remains unknown. In this study, we aim to investigate the therapeutic effect and the underlying mechanisms of HGWD against PIPN with pharmacological experiment and network pharmacology.

**Methods:**

Male Wistar rats were used to establish an animal model of PIPN and treated with different doses of HGWD for 3 weeks. Mechanical allodynia, thermal hyperalgesia and body weight were measured to evaluate the therapeutic effect of HGWD on PIPN rats. On the day of the sacrifice, blood, DRGs, sciatic nerve, and hind-paw intra-plantar skins were collected to assess neuroprotective effect of HGWD on PIPN. Next, network pharmacology was performed to decipher the potential active components and molecular mechanisms of HGWD, as were further verified by western blotting analyses in PIPN rats. Finally, the effect of HGWD on the chemotherapeutic activity of paclitaxel was evaluated in vitro and in vivo.

**Results:**

In rats with PIPN, HGWD reversed mechanical allodynia, thermal hyperalgesia, and ameliorated neuronal damage. Moreover, HGWD significantly increased the level of nerve growth factor, dramatically reduced IL-1β, IL-6, TNF-α levels and oxidative stress. Network pharmacology analysis revealed 30 active ingredients in HGWD and 158 candidate targets. Integrated pathway analysis identified PI3K/Akt and toll-like receptor as two main pathways responsible for the neuroprotective effect of HGWD. Further experimental validation demonstrated that HGWD expectedly inhibited the protein expression of TLR4, MyD88, IKKα, and p-NF-κB, and promoted PI3K, p-Akt, Nrf2, and HO-1 level in dorsal root ganglia. Last but not least, HGWD did not interfere with the antitumor activity of paclitaxel both in in vitro and in vivo models.

**Conclusion:**

These combined data showed that HGWD could inhibit paclitaxel-evoked inflammatory and oxidative responses in peripheral nervous system viaTLR4/NF-κB and PI3K/Akt-Nrf2 pathways involvement. The neuroprotective property of HGWD on PIPN provides fundamental support to the potential application of HGWD for counteracting the side effects of paclitaxel during chemotherapy.

**Supplementary Information:**

The online version contains supplementary material available at 10.1186/s13020-021-00488-1.

## Background

Chemotherapy remains a widely used and important treatment for cancer. However, nearly 60% of cancer survivors receiving chemotherapy would experience serious chemotherapy-induced peripheral neuropathy (CIPN), characterized with pain, tingling, and numbness in the hands and/or feet [1]. To date, no definite therapeutic strategy is available for CIPN prevention or management [2–5], making it an urgent need to discover effective pharmacological agents against CIPN.

Paclitaxel (PTX) is a well-known anticancer agent because of its high effectiveness against solid tumors. Since its favourable curative effect, PTX could result in severe peripheral neuropathy, affecting up to 50% of cancer patients [2]. PTX-induced peripheral neuropathy (PIPN) often requires a reduction in PTX dosage or even discontinuation of chemotherapy, seriously impairing the survival of cancer patients. Nonetheless, the unified mechanism of PIPN has not been fully elucidated thus far. According to recent research, PIPN may involve the loss of intra-epidermal nerve fibres (IENFs), mitochondrial damage, activation of transient receptor potential channel (TRP), and mitogen-activated protein kinase (MAPK). Oxidative stress and inflammation have also played a pivotal role in the development of a PIPN [6, 7]. Increased oxidative stress in peripheral sensory neurons can induce spontaneous discharge of neurons, and cause neuronal sensitisation and hyperalgesic responses [8]. Antioxidants, such as alphalipoic acid, have shown promising effects against CIPN via Nrf2 activation and subsequent oxidative stress inhibition [9]. Additionally, inflammation has been proven to be common in PIPN. Toll-like receptor 4/NF-κB (p65) signalling promotes the production of pro-inflammatory factors, which can damage neuron, suppress synaptic transmission, and enhance neuron response to noxious stimuli [10]. Furthermore, inflammation can activate multiple pathways, such as oxidative stress, protein kinase C, MAPK, and TRP, thereby inducing neuropathic pain. Direct inhibition of the TLR4/NF-κB pathway reduces reactive oxygen species (ROS) production and attenuates chemotherapy-induced neurotoxicity [11]. Despite advances in the mechanisms, PIPN management is still challenging, prompting researchers to seek for alternative treatment options.

**Fig. 1:**
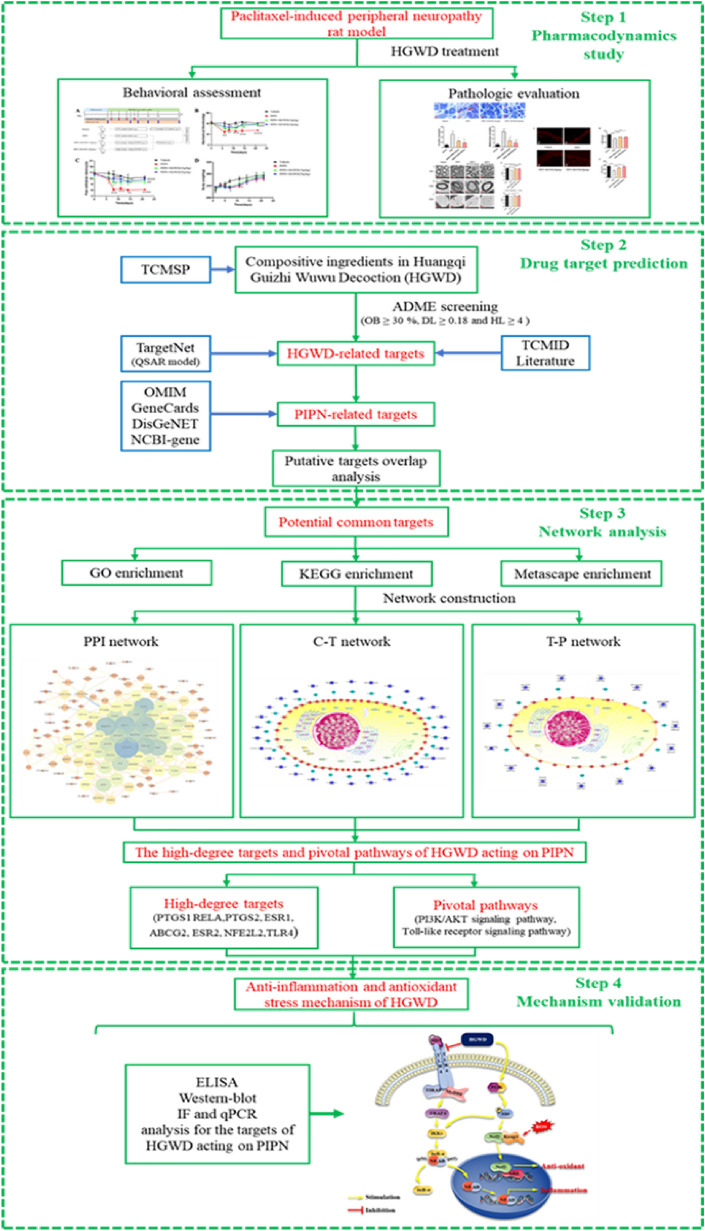
The research scheme of this study

Huangqi Guizhi Wuwu decoction (HGWD) is an herbal formula recorded in the “Golden Chamber”, comprising five crude drugs (Astragali Radix, Cinnamomi Ramulus, Paeoniae Radix Alba, Zingiberis Rhizoma Recens, and Jujubae Fructus). This prescription has long been used to control pain and numbness in stroke sequelae, peripheral nerve injury, and other diseases. Recent studies and our work suggest that HGWD is therapeutically effective for diabetic and oxaliplatin-induced peripheral neuropathy [12, 13]. HGWD improved neurological function, increased blood circulation and alleviated oxaliplatin-induced mechanical and cold hypersensitivity, suggesting that HGWD may offer potential opportunities to CIPN treatment. However, the effect and underlying mechanisms of HGWD against PIPN remains unknown, thus requiring further study. In this study, we integrated pharmacological evaluation and network pharmacology to decipher the efficacy and molecular mechanism of HGWD in PIPN treatment. The research framework of this study is shown in Fig. 1.

## Methods

### Animals

Thirty-two male Wistar rats (200 ± 30 g) were kept in a 12-h light/dark cycle with ad libitum access to water and food. The current protocol was approved by the Animal Care and Use Committee of Affiliated Hospital of Integrated Traditional Chinese and Western Medicine, Nanjing University of Chinese Medicine. All experimental procedures were conducted strictly in accordance with the Guide for Care and Use of Laboratory Animals.

### Drugs and chemicals

PTX was purchased from Shanghai Tauto Biotech Co., Ltd. (Shanghai, China). Crude drugs, including Astragali Radix (AR), Cinnamomi Ramulus (CR), Paeonia Radix Alba (PRA), Jujubae Fructus (JF), and Zingiberis Rhizoma (ZR) were provided by Jiangsu Hospital of Integrated Traditional Chinese and Western Medicine, and authenticated based on the instructions recorded in the Chinese Pharmacopoeia (2015 edition). The voucher specimens were deposited in Affiliated Hospital of Integrated Traditional Chinese and Western Medicine, Nanjing University of Chinese Medicine.

### HGWD preparation

HGWD was prepared in the following procedure: AR (140 g), CR (70 g), PRA (70 g), JF (70 g), and ZR (70 g) were immersed in distilled water (10 times the total weight) for 0.5 h and then gently refluxed two times, each time for 1 h. The two liquid extracts were combined and concentrated to 2 g/ml under reduced pressure and then stored at − 80 °C before use.

### Experimental design and treatment protocol

**Fig. 2:**
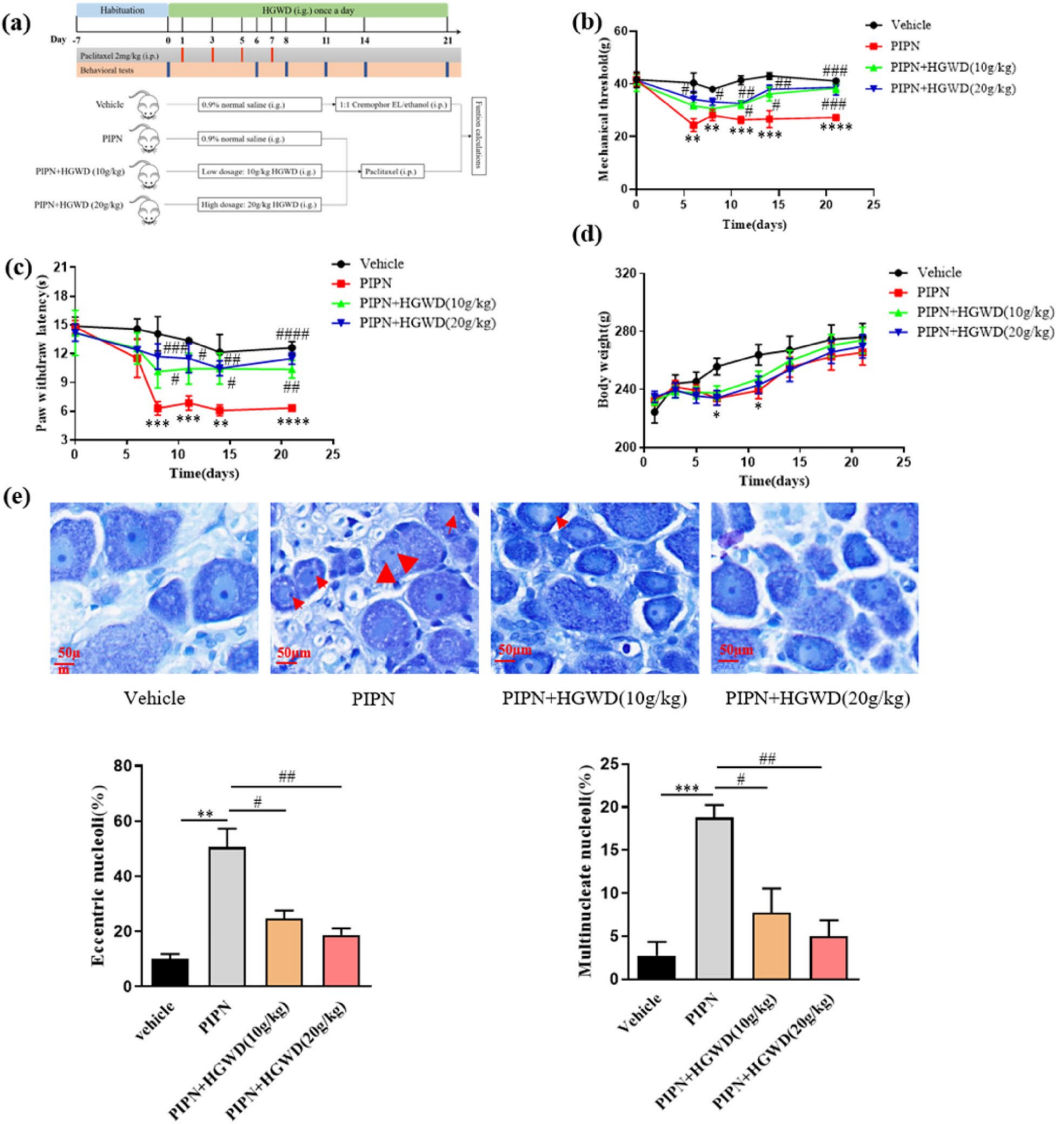
HGWD attenuated mechanical allodynia, thermal hyperalgesia, and morphological changes of DRG in PIPN rats. **a** Protocol showing drug administration and behavioral tests in the rat model. **b** Mechanical withdrawal threshold was measured by the von Frey test. **c** Thermal withdrawal threshold was evaluated by the hot plate test. **d** Time course effect of HGWD on body weight of rats. **e** DRG sections were stained by Nissl and observed by light micrographs (80 ×). The occurrence of eccentric nucleoli (red arrow) and multinucleolated neurons (red arrowhead) were analyzed. Histograms show the effect of HGWD on the incidence of nuclear and nucleolar pathological features. ^*^P < 0.05, ^**^P < 0.01 ^***^P < 0.001, vs. vehicle group; ^#^*P* < 0.05, ^##^*P* < 0.01, ^###^*P* < 0.001, vs. PIPN group, n = 8 rats/group

A schematic of the experimental design is shown in Fig. 2a. Rats were randomly divided into four groups (n = 8). PTX (2 mg/kg, prepared in cremophor EL/ethanol at a ratio of 1:1) was injected intraperitoneally (i.p.) for four alternate days (days 1, 3, 5, and 7) to induce PIPN, as described previously [11]. As the same dose of our previous study [13], HGWD 10 or 20 g/kg was administered orally once a day for 3 weeks from the first dose of PTX injection (1 h prior to each PTX injection, if coinciding with chemotherapy treatment). Behavioural tests were performed before and on days 6, 8, 11, 14, and 21 after the first PTX withdrawal. Blood and tissues were collected within 24 h after the final dose.

### Mechanical allodynia (von Frey test)

Mechanical allodynia was evaluated using von Frey filaments (Aesthesio, DanMic Global, LLC, USA) according to a previously reported method [13]. Rats were placed individually in small cages with a mesh floor for 10 min. A monofilament was applied perpendicularly to the plantar surface of the hind paw, delivering a constant pre-determined force for 2–5 s [14]. If the rat presented a positive response (withdraws or licks the paw), then the minimum force required to elicit a clear paw withdrawal or shaking was recorded. Measurements were repeated three times at 5-min intervals, and the mean value was calculated.

### Thermal hyperalgesia (hot-plate test)

The hot plate test was used to determine heat thresholds of rat. A rat was placed on a metal surface maintained at a constant temperature (50–55 ℃) [14]. The response latency, which is the time taken to observe nocifensive behaviors (hind paw withdrawal or licking, stamping), was recorded. According to the results of our pre-experiment results, thermal hyperalgesia was assessed at 52 °C. A cut-off time of 30 s was applied to avoid tissue damage.

### Histological analysis of dorsal root ganglia (DRG) and sciatic nerve tissue

The dissected lumbar 4–6 (L4-6) DRG was fixed in 10% formalin solution, embedded in paraffin, and then sliced into 4–5 μm-thick sections. The sections were then stained with Nissl staining solution and examined microscopically. Sciatic nerve samples were harvested, fixed in 4% glutaraldehyde, and post-fixed in 1% osmic acid at 25 °C for 2 h. Samples were then dehydrated with graded ethanol series and embedded in Epon 812. Ultrathin sections (80 nm) were prepared, double-stained with uranium acetate and lead citrate, and then observed with an HT7700 transmission electron microscope (Hitachi, Tokyo, Japan).

### Quantification of IENF density

For quantification of IENFs, hind-paw intra-plantar skins of rats were collected for immunohistochemical studies. The antibodies used were anti-protein gene product 9.5 (PGP9.5) primary Ab (ab108986, 1:200; Abcam, Cambridge, MA, USA) and Cy3-conjugated goat anti-rabbit IgG (ab6939, 1:300; Abcam). PGP9.5-positive IENFs were counted and expressed as the numbers of fibres/length of epidermis (IENFs/mm). The scorer was completely blinded to the experimental setup.

### Measurement of nerve growth factor (NGF) and oxidative stress markers

Serum levels of NGF and markers of oxidative stress, such as superoxide dismutase (SOD), malondialdehyde (MDA), and 8-isoprostane F2α, were measured. MDA and SOD levels were examined by colorimetric kits (Beyotime Biotechnology Co.Ltd, Shanghai, China). NGF and 8-isoprostane F2α levels were measured using a rat beta-NGF ELISA Kit (Invitrogen, Grand Island, NY, USA) and 8-iso-PGF2α ELISA kit (Enzo Life Sciences, New York, USA), respectively. Catalog numbers and assay ranges of ELISA kits are listed in Additional file 1: Table S1.

### Inflammatory factors measurement

The serum levels of inflammatory cytokines, including interleukin (IL)-1β, IL-6, tumor necrosis factor-α (TNF-α), and IL-10 were tested using ELISA kits (Multi Sciences (Lianke) Biotech Co., Ltd., Hangzhou, China). IL-1β, IL-6, TNF-α, and IL-10 mRNA in DRG tissue was measured by real-time polymerase chain reaction (RT-PCR). The gene-specific primers for rat IL-1β, IL-6, TNF-α, and IL-10 are listed in Additional file 1: Table S4.

### Construction of the chemical component database and screening of active compounds with its targets

All chemical ingredients in HGWD were collected from the TCMSP and TCMID databases. The TCMSP database is a systematic pharmacology platform that reveals the mechanisms of Tradition Chinese Medicine by identifying a drug-target network, covering pharmacokinetic properties including absorption, distribution, metabolism, and excretion (ADME). In the present study, components with oral bioavailability (OB) ≥ 30%, drug likeness (DL) ≥ 0.18, and drug half-life (HL) ≥ 4 were selected as active ingredients possessing biological activity for further study. The TCMID database was applied to replenish HGWD ingredients according to target proteins that were not recorded in the TCMSP database. Next, to reveal the pharmacological mechanism of the active ingredients of HGWD, the potential targets of the candidate active compounds were predicted using the QSAR model in TargetNet. The target information collected from TCMSP, TCMID, and DrugBank databases and literatures were integrated to supplement the target database.

### Construction of “HGWD-PIPN” common targets database

The main targets of PIPN-related diseases were obtained from the following databases: OMIM, GeneCards, NCBI-gene, and DisGENET. In this study, “paclitaxel-induced peripheral neuropathy” was used as the main keyword to screen disease-related targets in the above bioinformatics websites. Next, we mapped the prediction targets of the HGWD with PIPN-related disease targets. Both the PIPN-related targets and HGWD-related targets obtained were imported to draw a Venn diagram, and the overlapping part showed the common targets of diseases and compounds. In addition, the common targets network was established and inspected using the Cytoscape 3.7.2 software.

### Gene Ontology (GO), Kyoto Encyclopaedia of Genes and Genomes (KEGG), and target proteins distribution enrichment analyses

GO and KEGG pathway enrichment analyses were carried out using the KOBAS website. Enrichment analysis for cell and tissue distribution of the target proteins of HGWD was conducted on the Metascape website. To elucidate whether HGWD exerts neuroprotective effect in chemotherapy, we subjected the candidate targets to enrichment analysis of biological processes, cellular components, molecular functions, and signalling pathways, with *P*-value < 0.05 considered as statistically significant. The results were mainly visualised using the ggplot2 package in R 3.6.0. Information of Public databases and its website involved in the study are listed in Additional file 1: Table S2.

### Network construction

Protein–protein interaction (PPI) predictions were used to predict the outcome of pairs or groups of protein interactions. In this study, PPI network was generated by importing the common targets into the String database, with the species restricted to *“Homo sapiens”* and the highest confidence core > 0.9. Finally, the obtained results were exported in the TSV format and imported into the Cytoscape software for visualisation. To analyse and visualise the relationship between effective active compounds, targets, and pathways, the compound-target (C-T) network and target-pathway (T-P) network were established by Cytoscape 3.7.2 software.

### Western blotting

DRG tissues were harvested, washed, homogenised, and extracted using RIPA lysis buffer (Beyotime Biotechnology Co., Ltd.) containing protease inhibitor. Protein concentrations of lysates were measured by the bicinchoninic acid method (Beyotime Biotechnology Co., Ltd.). Protein samples were electrophoresed in a 10% SDS-PAGE gel and transferred to polyvinylidene difluoride membranes (Millipore, Billerica, MA, USA). The membranes were blocked with 5% non-fat milk and incubated with the following primary antibodies overnight at 4 °C: TLR4 (1:1000; Affinity Biosciences, Ltd., Cincinnati, OH, USA); MyD88, Keap1 (1:1000; Cell Signalling Technology, USA); NF-κB p65, p-NF-κB (1:1000; Absin Biotechnology Co., Ltd); PI3K, Akt, phospho-Akt(p-Akt) (1:1000; from Cell Signaling Technology, USA); Nrf2 (1:1000; Abcam); HO-1(1:1000, ABclonal Biotechnology Co., Ltd); and β-tubulin (1:3000; Affinity Biosciences, Ltd., Cincinnati, OH, USA). Afterwards, the membranes were washed with PBST, incubated with HRP-conjugated secondary antibodies (Affinity Biosciences, Ltd.), and visualised by enhanced chemiluminescence (Beyotime Biotechnology Co. Ltd.). The results were quantified using the NIH ImageJ software. Catalog numbers of antibodies in the western blotting test are listed in Additional file 1: Table S3.

### MTT assay

Cancer cell lines, including MDA-MB-231 and CFPAC-1 cells, were purchased from the Bank of Cell, Institute of Cell Biology, Chinese Academy of Sciences (Shanghai, China). Cells were maintained in DMEM (MDA-MB-231 cells) or IMEM (CFPAC-1 cells) plus 10% foetal bovine serum at 37 °C in 5% CO_2_. The effect of HGWD on the tumor cell-killing ability of PTX was evaluated via an enzymatic MTT assay.

### Tumor xenograft model

CFPAC-1 cells (4 × 10^6^) were injected subcutaneously into the right flank of nude BALB/c mice (aged 3–4 weeks). When the tumor had grown to approximately 150–300 mm^3^, the mice were randomised into three groups (n = 7 per group): vehicle (normal saline), PTX (10 mg/kg in normal saline, i.p., q.d (days 1, 3, and 5)), and PTX (10 mg/kg) combined HGWD (30 g/kg, i.g., q.d (days 1–22)). Tumor size was measured every 3 days and calculated according to the following formula: tumor volume = length × width^2^/2. At the end of the experiment, the mice were sacrificed, and tumor xenografts were excised and weighed.

### Statistical analysis

The difference between two different groups was analysed via unpaired Student’s *t*-test using the GraphPad Prism 8 software. A one-way analysis of variance (ANOVA) followed by Bonferroni analysis was used for in vivo comparisons among multiple groups. *P* < 0.05 was regarded as statistically significant.

## Results

### HGWD alleviates both mechanical allodynia and heat hyperalgesia in a rat model of PIPN

To examine the effect of HGWD on PIPN, we established a rat model of PIPN according to reported protocols [15]. A cumulative dose of 8 mg/kg PTX injection (4 × 2 mg/kg, 2 days apart, i.p.) robustly and persistently reduced hind-paw withdrawal threshold in rats (the PIPN group), compared with the vehicle (Fig. 2b , P < 0.01). PTX-induced mechanical allodynia was inhibited by HGWD administration. At day 14, paw-withdrawal threshold in the PIPN group was 26.7 ± 7.9 g, whereas that in HGWD (20 g/kg)-treated rats increased to 38.0 ± 4.6 g (Fig. 2b , P < 0.01). In rats receiving the vehicle, the threshold of withdrawal responses remained basically unchanged during the experimental period. Next, we measured the heat thermal nociceptive sensitivity of rats. The results showed that the baseline paw-withdrawal thermal latency was approximately 14.5 ± 0.3 s in each group before the treatment onset (Fig. 2c). No significant changes in paw-withdrawal latency were detected in rats receiving the vehicle. In contrast, PTX significantly decreased the heat thermal nociceptive threshold (from 14.8 to 6.3 s) at day 8 (*P* < 0.001). HGWD produced persistent relief of thermal sensitivity induced by PTX (Fig. 2c). However, HGWD administration could not reverse the loss of body weight after PTX treatment (Fig. 2d). These data indicated that HGWD could effectively counteract PTX-induced sensory disturbances.

### HGWD attenuates PTX‑induced histological changes in DRG and sciatic nerves

Next, we determined whether functional changes in peripheral neurons following PTX treatment were accompanied by structural alterations in peripheral nervous tissue. Histological examination of DRG revealed that the cytoplasm of neurons and satellite cells appeared normal in all experimental groups. Compared with those in vehicle group, DRG neurons in PTX-treated rats exhibited increased incidence of multinucleolate cell bodies (Fig. 2e arrowheads) and eccentric nucleoli (Fig. 2e red arrow). In HGWD-treated groups, the percentages of multinucleated nuclei and nucleolar segregation were drastically reduced compared with those in rats treated with PTX alone (Fig. 2e).

**Fig. 3:**
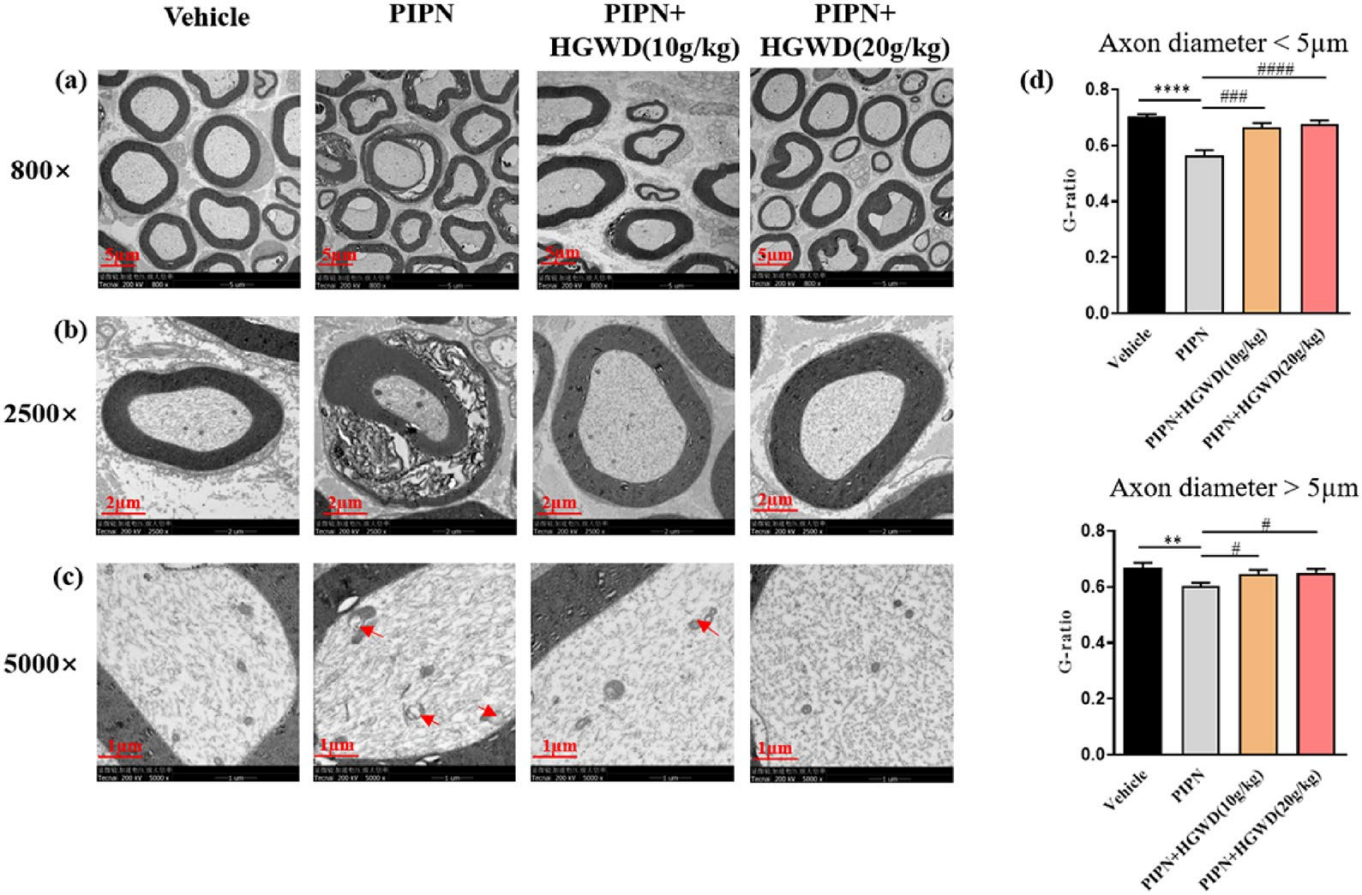
HGWD attenuated paclitaxel-induced histological changes in sciatic nerves. **a** Cross view of ultrastructure of rat sciatic nerve. Scale bar, 5 μm. **b** Paclitaxel-induced sciatic nerve damage includes axonal degeneration and myelin damage. Scale bar, 2 μm. **c** Representative transmission electron microscopy images show that paclitaxel caused swollen and vacuolated mitochondria (red arrow). Scale bar, 1 μm. **d** G-ratio (the ratio of inner axon circumference to outer myelin circumference) measurement in the sciatic nerves. Each value represents mean ± SEM of at least 32 sciatic nerves. ^**^P < 0.01, ^****^P < 0.0001, vs. vehicle group; ^#^*P* < 0.05, ^###^*P* < 0.001, ^####^*P* < 0.0001, vs. PIPN group

Electron microscopic examination of sciatic nerves showed the characteristic appearance of ultrastructural damage after PTX treatment. As shown in Fig. 3a and b, PTX clearly induced axonal swelling, altered myelination, and the loosening and breakdown of myelin sheath in sciatic nerves. Moreover, PTX caused swollen and vacuolated mitochondria in the axon (Fig. 3c, red arrow). G-ratio, a key measure of myelination integrity, was significantly lower in both the large (axon diameter > 5 μm) and small (axon diameter < 5 μm) fibres of sciatic nerves in the PIPN group (0.60 ± 0.05; 0.56 ± 0.09) than in the vehicle group (0.66 ± 0.08; 0.7 ± 0.04) (Fig. 3d). HGWD significantly rescued PTX-induced neurotoxic effects in sciatic nerve, including axonal degeneration and myelin damage.

### HGWD protects against PTX‑induced IENF loss and reduction of NGF levels

**Fig. 4:**
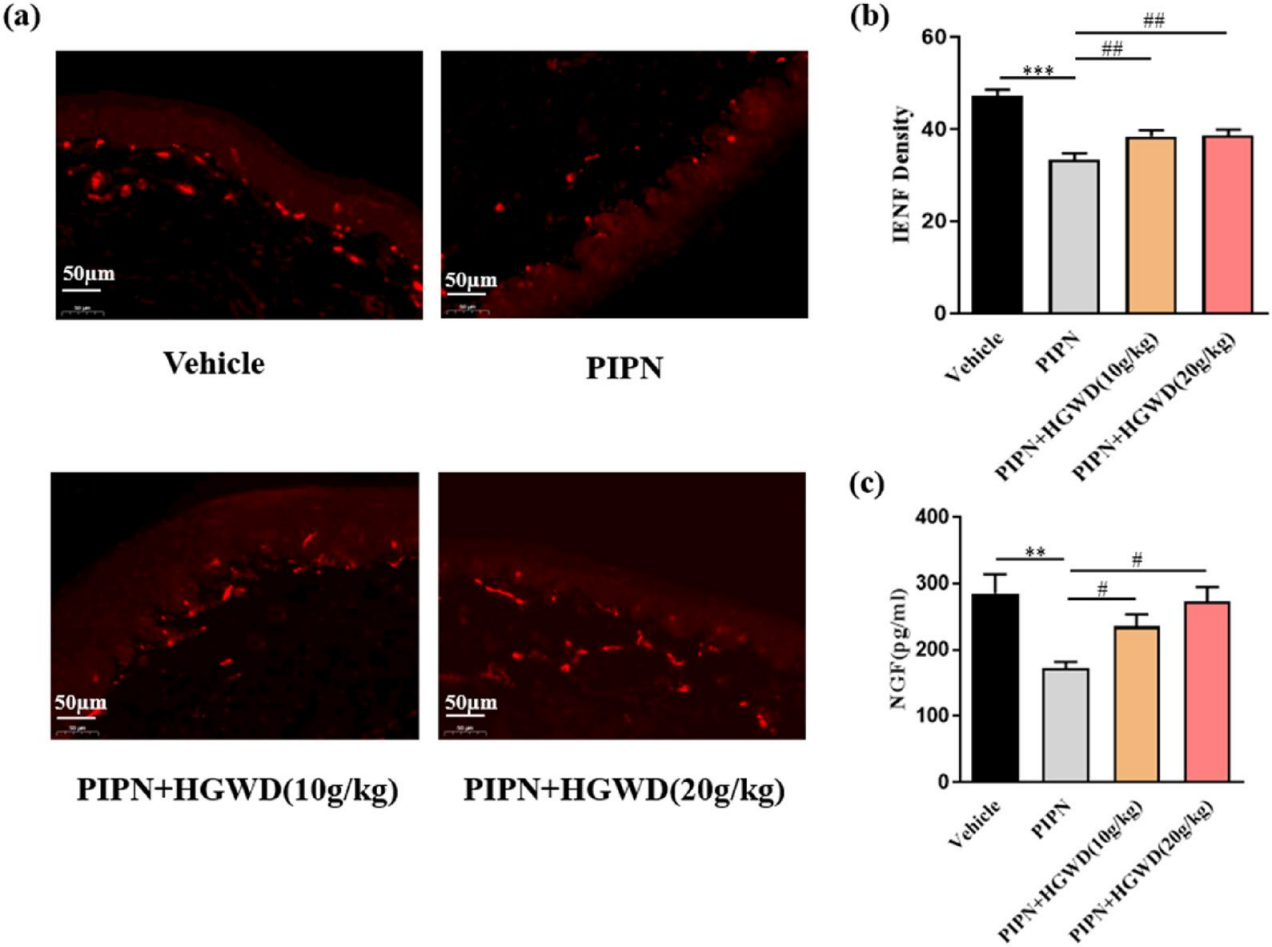
HGWD-treated rats have reduced paclitaxel-induced IENF loss and increased NGF level. **a** IENF in the hind paws of rats were identified by PGP 9.5 staining (red). Representative images were presented. **b** Quantification of the density of IENF. Scale bars: 50 μm. **c** Serum NGF level in each group. ^**^P < 0.01, ^***^P < 0.001, vs. vehicle group; ^#^*P* < 0.05, ^##^*P* < 0.01, vs. PIPN group, n = 8 rats/group

IENFs, projecting from the DRG or trigeminal nerve, are regarded to be responsible for sensation and noxious impulse transmission. As an early sign of PIPN, IENF loss in the plantar surface of the hind paws is associated with hypersensitivity. To explore whether HGWD alleviate PTX-induced IENF loss, the density of IENFs in the hind paws of rats was analysed with neuronal marker PGP9.5. As shown in Fig. 4a, b, PTX injection significantly reduced the density of nerve fibres entering the epidermis, compared with that in vehicle-treated rats. After HGWD administration, PTX-induced IENF retraction was significantly suppressed.

NGF, a nerve growth-regulating factor secreted mainly by neurons or glial cells, is correlated with the occurrence and severity of PIPN. Decreased NGF expression and transport have been well illustrated in the development of CIPN [16]. As illustrated in Fig. 4c, serum NGF level was remarkably reduced after PTX treatment compared with that after vehicle treatment (171.7 ± 20.5 versus 285.1 ± 51.66 pg/ml, respectively; *P* < 0.01). In HGWD (20 g/kg)-treated group, the reduction was considerably lower (272.1 ± 39.1 pg/ml; *P* < 0.05) than that in the PIPN model group, showing no significant difference from that in the vehicle group (*P* > 0.05). These results suggest that HGWD can increase serum NGF levels and may promote neuron repairing.

### HGWD reduces inflammatory responses in PTX-treated rats

**Fig. 5:**
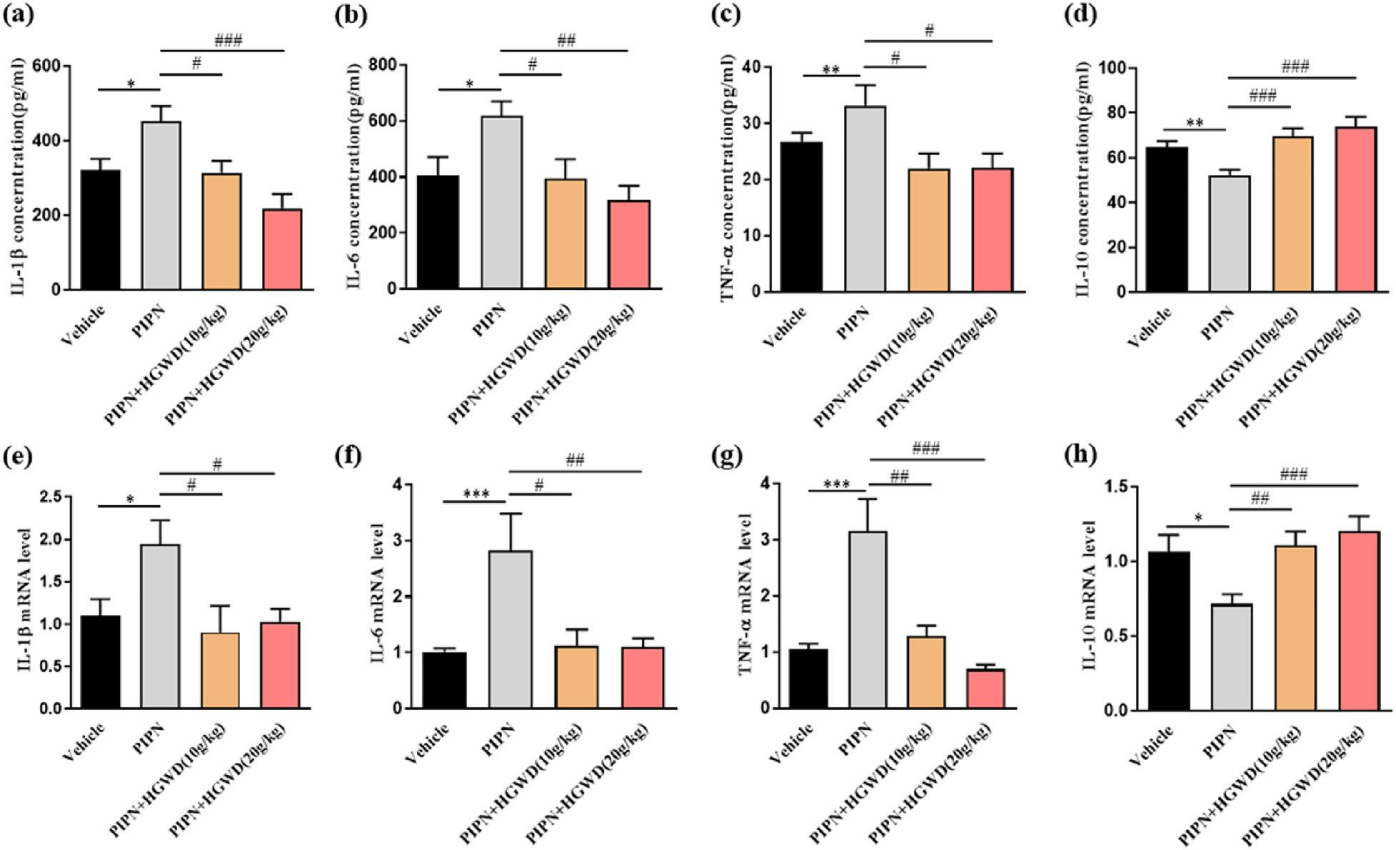
HGWD alleviated paclitaxel-induced inflammation responses. **a–d** Protein expression of proinflammatory cytokines in serum were determined by ELISA. **e–h** mRNA expression of proinflammatory cytokines in DRG neurons were detected by RT-PCR. ^**^P < 0.01 vs. vehicle, ^##^*P* < 0.01 vs. PIPN alone

Inflammation response has been regarded as an important trigger of CIPN. In this study, serum IL-1β, IL-6, and TNF-α levels were significantly upregulated after PTX administration [IL-1β and IL-6, *P* < 0.05; TNF-α, *P* < 0.01), but this increase was markedly inhibited in both groups treated with high- and low-dose HGWD (Fig. 5a–c)]. IL-10, an active anti-inflammatory cytokine that suppresses pro-inflammatory signals, was found to be increased in HGWD-treated rats, suggesting the anti-inflammatory effect of HGWD (Fig. 5d). A similar pattern was observed for IL-1β, IL-6, TNF-α, and IL-10 mRNA in rat DRG (Fig. 5e–h). These data suggest that HGWD could markedly inhibit the inflammatory response evoked by PTX.

### HGWD inhibits oxidative stress in PTX-treated rats

**Fig. 6:**
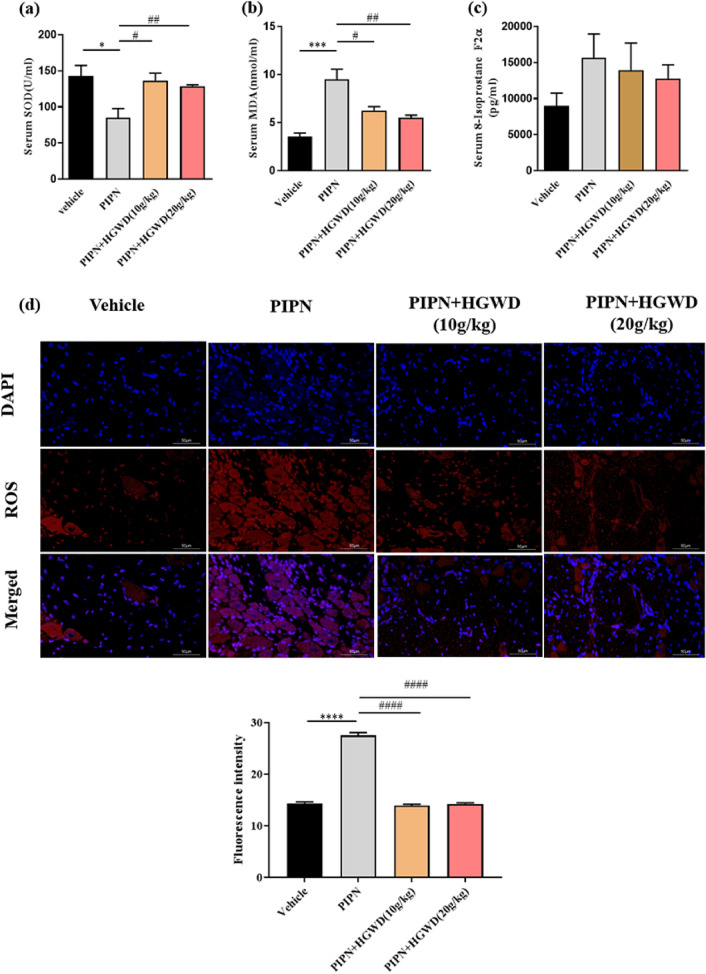
HGWD reduced oxidative stress in PTX-treated rats. **a** SOD activity. **b** MDA content. **c** 8-isoprostane F2α level. **d** ROS production detected with immunofluorescence in DRG. Results are the mean ± SEM from three independent experiments. ^*^P < 0.05, ^***^P < 0.001 vs. vehicle group, ^#^*P* < 0.05, ^##^*P* < 0.01 vs. PIPN group

Excessive oxidative stress has been found to be involved in the development of CIPN. The levels of oxidative stress parameters (SOD, MDA, 8-isoprostane F2α, and ROS) were detected in this work. Compared with the vehicle group, the serum of PTX-treated rats showed increased MDA of and decreased SOD (Fig. 6a–c). HGWD dramatically reduced the elevated levels of MDA and promote SOD activity (*P* < 0.05 or *P* < 0.01). In the HGWD (20 g/kg)-treated group, elevated 8-isoprostane F2α level was obviously reduced (from 15.7 ± 5.7 to 12.7 ± 3.4 ng/ml). To further elucidate the antioxidative effect of HGWD, ROS level in DRG was also measured (Fig. 6d). The results revealed that PTX increased ROS generation in DRG neurons, whereas HGWD blocked ROS production. These findings indicate that HGWD inhibits oxidative stress and maintains a redox balance in PTX-treated rats.

### Active compounds screening and target prediction of HGWD formula

**Fig. 7:**
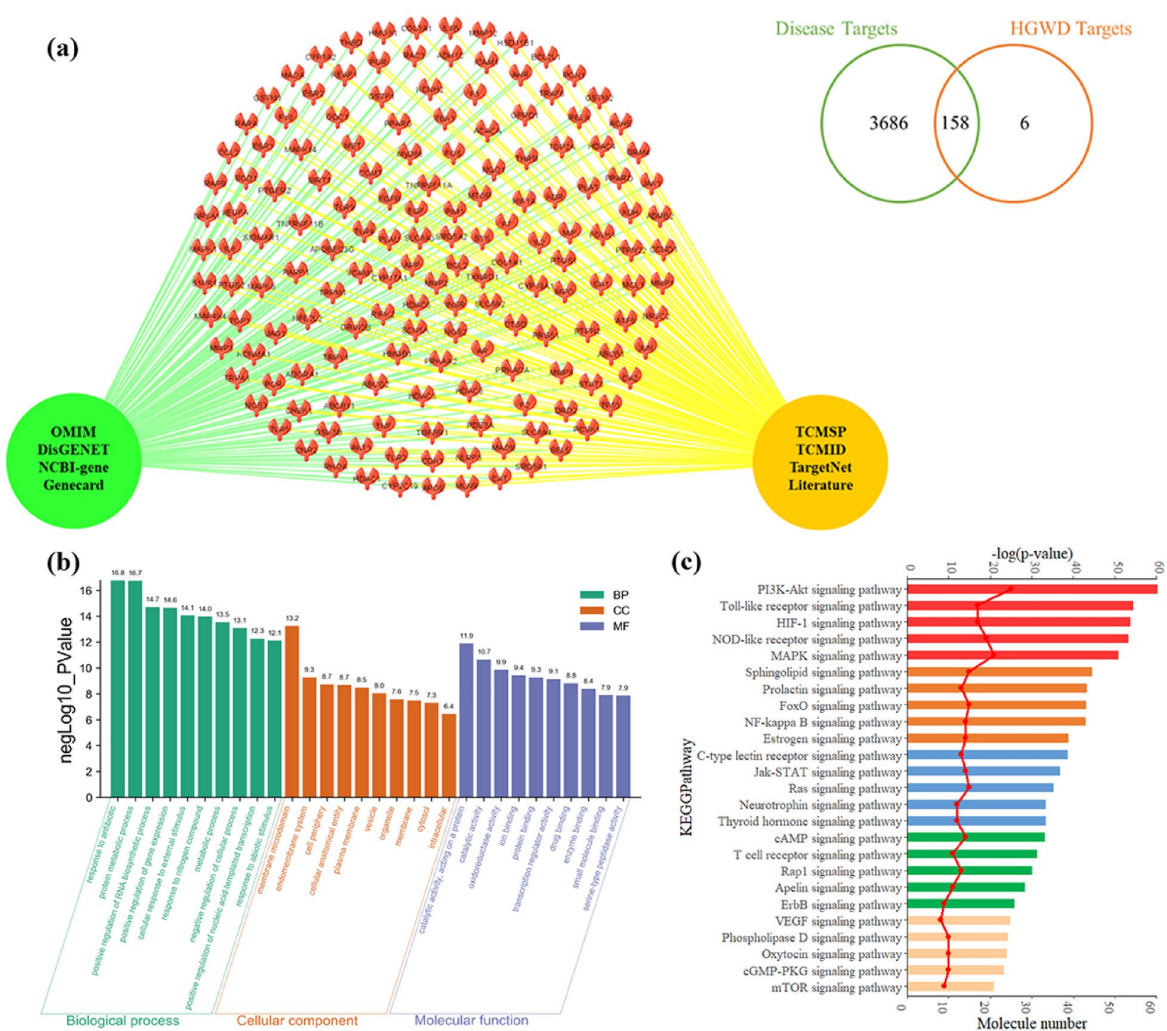
Target prediction of HGWD with GO and KEGG enrichment analysis. **a** The Venn diagram of disease targets and HGWD targets showed 158 key target proteins in the overlapping part, and the schematic diagram was displayed. **b** The top 10 significant terms respectively in GO biological process, cellular component and molecular function classification, and the order of importance was ranked from left to right by -log(p-value). **c** The top 25 significant terms in KEGG pathways enrichment. The molecule number of target genes enriched in the KEGG pathway was showed, and the order of importance was ranked from top to bottom by -log(p-value)

In this study, active chemical compounds in HGWD were searched from the TCMSP and TCMID databases, and the ingredients and their physicochemical properties were collected. Next, an ADME system was applied to filter out the potential active compounds of HGWD, which met the standards of OB ≥ 30%, DL ≥ 0.18, and HL ≥ 4. Finally, a total of 30 active chemical ingredients were obtained from HGWD (Table 1). These active compounds mainly belonged to phenols, flavonoids, and lignans. To elucidate the action mechanism of HGWD, the possible targets of active compounds were clarified by SMILES, and predicted using the TargetNet database and literature mining. The main targets of PIPN-related diseases were obtained from several databases, including OMIM, NCBI-gene, GeneCards, and DisGENET. By mapping the prediction targets of HGWD with PIPN-related disease targets, 158 potential common targets were obtained and displayed in the overlapping part from the Venn diagram and the interactive network (Fig. 7a).

**Table 1:** Active compounds and their corresponding parameters in HGWD formula

No	CAS	Compounds	Herb	Degree	Structure
1	480-18-2	Taxifolin	CR/ AR	13	
2	486-39-5	(S)-Coclaurine	JF	13	
3	470-82-6	1,8-cineole	ZR	10	
4	99-50-3	3,4-dihydroxybenzoicacid	CR	20	
5	N/A	3,9-di-O-methylnissoln	AR	11	
6	77398-90-4	4-gingerol	ZR	1	
7	23513-14-6	6-gingerol	ZR	21	
8	555-66-8	6-shogaol	ZR	17	
9	2086-83-1	Berberine	JF	19	
10	20575-57-9	Calycosin	AR	20	
11	104-55-2	Cinnamaldehyde	CR	25	
12	N/A	Cinnamic acid	CR	23	
13	N/A	Coumestrol	JF	15	
14	485-72-3	Formononetin	AR	38	
15	130-86-9	Fumarine	JF	14	
16	465-99-6	Hederagenin	AR	10	
17	480-19-3	Isorhamnetin	AR	31	
18	3301-49-3	Jaranol	AR	15	
19	520-18-3	Kaempferol	AR/ PRA	45	
20	32383-76-9	Medicarpin	AR	3	
21	117-39-5	Quercetin	AR/ JF	69	
22	92-61-5	Scopoletin	JF	15	
23	2810-21-1	Stepharine	JF	11	
24	16562-13-3	Stepholidine	JF	3	
25	83-48-7	Stigmasterol	AR/ZR/ JF	17	
26	122-48-5	Zingerone	ZR	11	
27	83-46-5	Beta-sitosterol	CR/ ZR/ PRA/ AR/ JF	22	
28	23180-57-6	Paeoniflorin	AR/PRA	14	
29	472-15-1	Betulic acid	PRA/ AR/ JF	2	
30	73536-69-3	Bifendate	AR	6

### GO, KEGG, and target proteins distribution enrichment analyses

After identifying the potential common targets of HGWD and PIPN, GO and KEGG enrichment analyses were conducted to explore the action mechanism of HGWD. As shown in Fig. 7b and c, the biological processes, cellular component, and molecular function categories in GO enrichment (*P* < 0.05) and the signal pathways in KEGG enrichment (*P* < 0.05) were screened and visualised. GO enrichment results showed that the targets of HGWD involved 10 significant terms in biological processes, cellular components, and molecular functions. Among them, the closely related biological processes included RNA biosynthetic process, positive regulation of gene expression, and nucleic acid-templated transcription. KEGG enrichment analysis showed that 25 pathways were involved in the protective effect of HGWD against PIPN. The top 3 pathways ranked by −log(p-value) were as follows: the PI3K-Akt, Toll-like receptor, and HIF-1 signalling pathway. Enrichment analysis of 158 potential common target proteins showed that the cell and tissue distribution of the potential target proteins mainly included the liver, smooth muscle, spleen, and DRG (Additional file 1: Figure S1).

### PPI network analysis

**Fig. 8:**
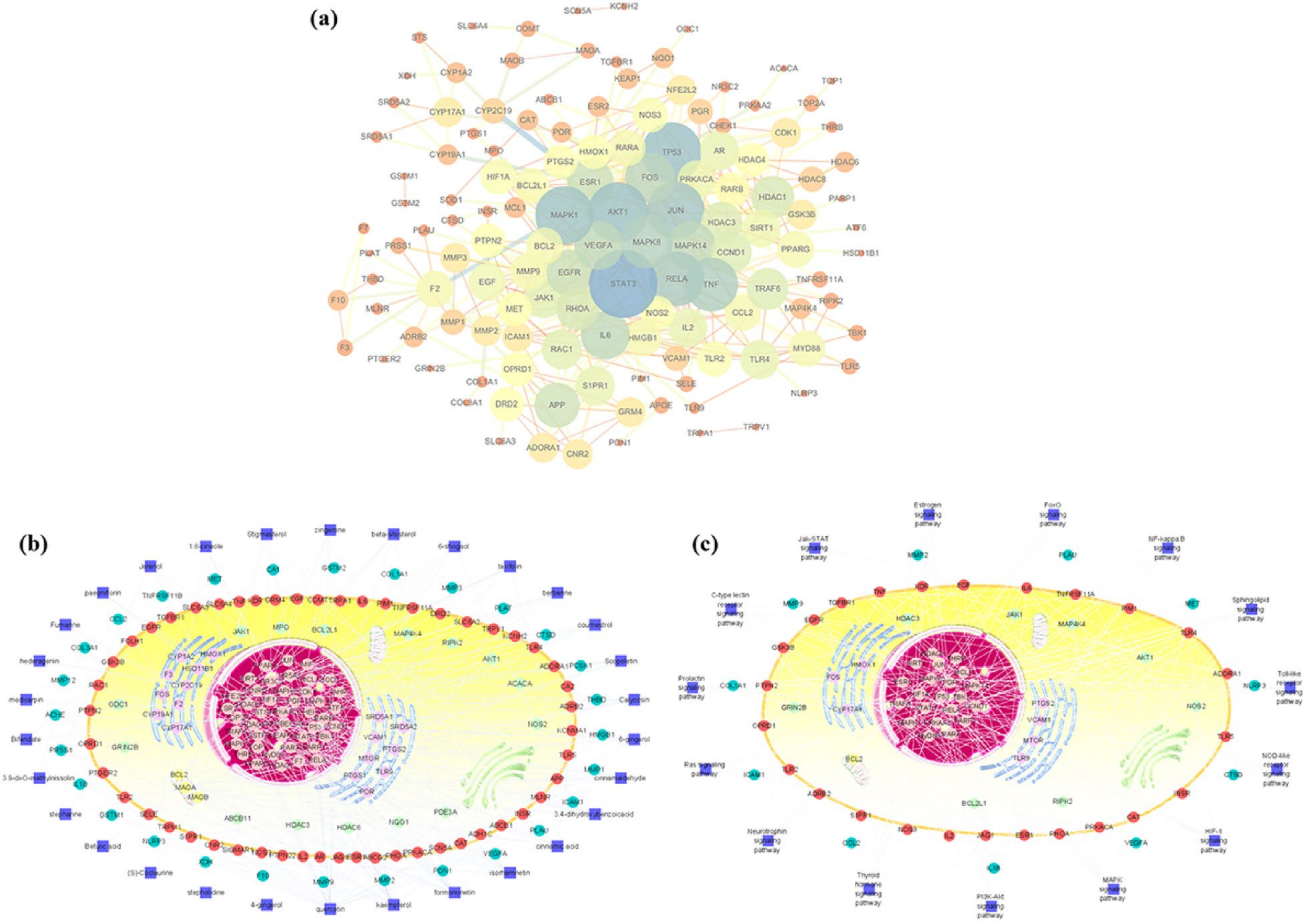
Network analysis**. a** Protein–protein interaction network analysis. The thickness of the line reflects the magnitude of Betweenness Centrality, and the color and depth of the nodes (blue → yellow → red) represent the size of the degree value (high → low). **b** Compound-Target network analysis. The navy-blue squares represent the compounds, and diverse colors of circles represent the target proteins distributed in various areas of the cell, such as extracellular region, plasma membrane, cytosol, endoplasmic reticulum, mitochondrion, and nucleus. **c** Target-Pathway network analysis. The navy-blue squares represent the pathways which were the top 15 significant changes in KEGG enrichment

PPI network was built to analyse the complex interactome organisation between potential common targets. As shown in Fig. 8a, the thickness of the line reflects the magnitude of Betweenness Centrality, and the color and depth of the nodes (blue → yellow → red) represent the size of the degree value (high → low). Commonly, proteins with a high degree are considered hubs, which should be focused on and regarded as core targets in network analysis. The results revealed that the degree values of STAT3, AKT1, TP53, MAPK1, JUN, RELA, TNF, and MAPK8 were greater than triple the average degree value (average degree = 7.19). These targets with a high degree value played an important role in the PPI network and may be of great significance in the treatment of PIPN by HGWD.

### C-T and T-P network analysis

The C-T network diagram was constructed based on 188 nodes (30 potential compounds and 158 potential targets) and 535 edges (Fig. 8b). Quercetin is the pivotal compound of HGWD, showing the highest number of target interactions (degree = 69), followed by kaempferol (degree = 45), formononetin (degree = 38), isorhamnetin (degree = 31), cinnamaldehyde (degree = 25), and cinnamic acid (degree = 23). In addition, the high-degree targets were related to multiple compounds, such as PTGS1 (degree = 20), RELA (degree = 19), PTGS2 (degree = 17), ESR1 (degree = 16), ABCG2 (degree = 13), NFE2L2 (degree = 12), and TLR4 (degree = 12). The T-P network consisting of 71 targets and 15 enriched pathways is shown in Fig. 8c. Many pathways are regulated by multiple target proteins, as might be the crucial pharmacological mechanism of HGWD against PIPN. The key signalling pathways included the PI3K/AKT (degree = 25), MAPK (degree = 21), NOD-like receptor (degree = 19), and Toll-like receptor (degree = 17) signalling pathway. These high-degree targets and pathways might be the major mediators of the neuroprotective effect of HGWD against PIPN.

### HGWD downregulates TLR4/NF-κB signalling and activates PI3K/Akt-Nrf2 pathway in PTX-treated rats

**Fig. 9:**
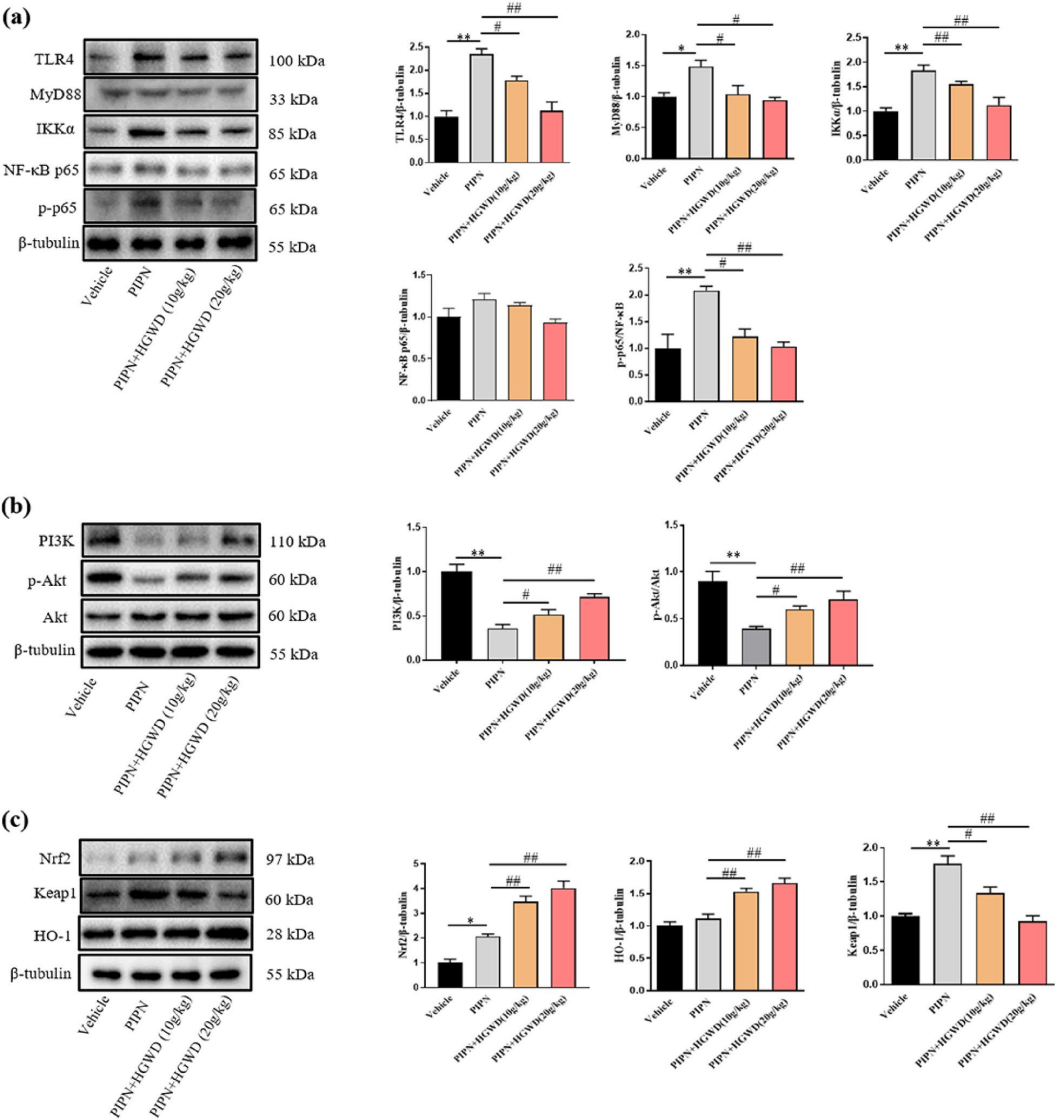
HGWD regulated the expression of TLR4/NF-κB and PI3K/Akt-Nrf2 pathways in PTX-treated rats. **a** HGWD downregulated TLR4/NF-κB signaling. **b** HGWD upregulated the expression of PI3K and phosphorylated Akt proteins. **c** HGWD activated Nrf2-OH-1 pathway. Levels of TLR4, MyD88, IKKα, NF- κB, p-NF-κB, PI3K, p-AKT, Nrf2, Keap1, HO-1, and β-tubulin from DRG tissues were determined by western blot analysis. Data are expressed as means ± SEM. ^*^P < 0.05, ^**^P < 0.01 ^***^P < 0.001, vs. vehicle group; ^#^*P* < 0.05, ^##^*P* < 0.01, ^###^*P* < 0.001, vs. PIPN group

To validate the results of the network pharmacology analysis and elucidate the mechanisms underlying the neuroprotective effect of HGWD, the expression of key proteins related with TLR4/NF-κB and PI3K/Akt-Nrf2 signalling pathways in DRG were validated by western blotting analysis. It was found that the expression of TLR4 and its adaptor protein MyD88 were significantly increased after PTX treatment (Fig. 9a). As TLR4/MyD88 pathway activates downstream effector NF-κB and accelerates pro-inflammatory responses, NF-κB and its core regulator IKKα were also analysed. As presented in Fig. 9a, p-NF-κB and IKKα was significantly elevated in the DRGs of PTX-treated rats. HGWD dramatically decreased TLR4, MyD88, IKKα, and p-NF-κB expression, indicating that HGWD suppressed the PTX-induced activation of TLR4/NF-κB signalling.

PI3K/Akt-Nrf2 pathway is a classical pathway regulating antioxidative stress. In this study, the activation of the PI3K/Akt pathway and the expression of Nrf2 after HGWD treatment were further evaluated to better understand how HGWD exerts protective effect against PIPN. As shown in Fig. 9b, the levels of PI3K protein and phosphorylated Akt in the DRG of PIPN rats were decreased compared with those in the vehicle group. Treatment of PIPN rats with 10 and 20 mg/kg HGWD increased PI3K protein levels and the downstream p-Akt/Akt ratio. The antioxidant transcription factor Nrf2 plays a pivotal role in defensive responses against oxidative stress. In our study, we found that the expression of Nrf2 and HO-1 was significantly elevated in the DRGs of the PIPN model group (Fig. 9c), suggesting the activation of the endogenous antioxidative defence response in PIPN rats. HGWD treatment further promoted the expression of Nrf2 and HO-1. These results suggest that HGWD may play an oxidative stress role by activating the PI3K/Akt-Nrf2 pathway.

### HGWD did not compromise the antitumor effect of PTX in cancer cells and mouse xenograft models

**Fig. 10:**
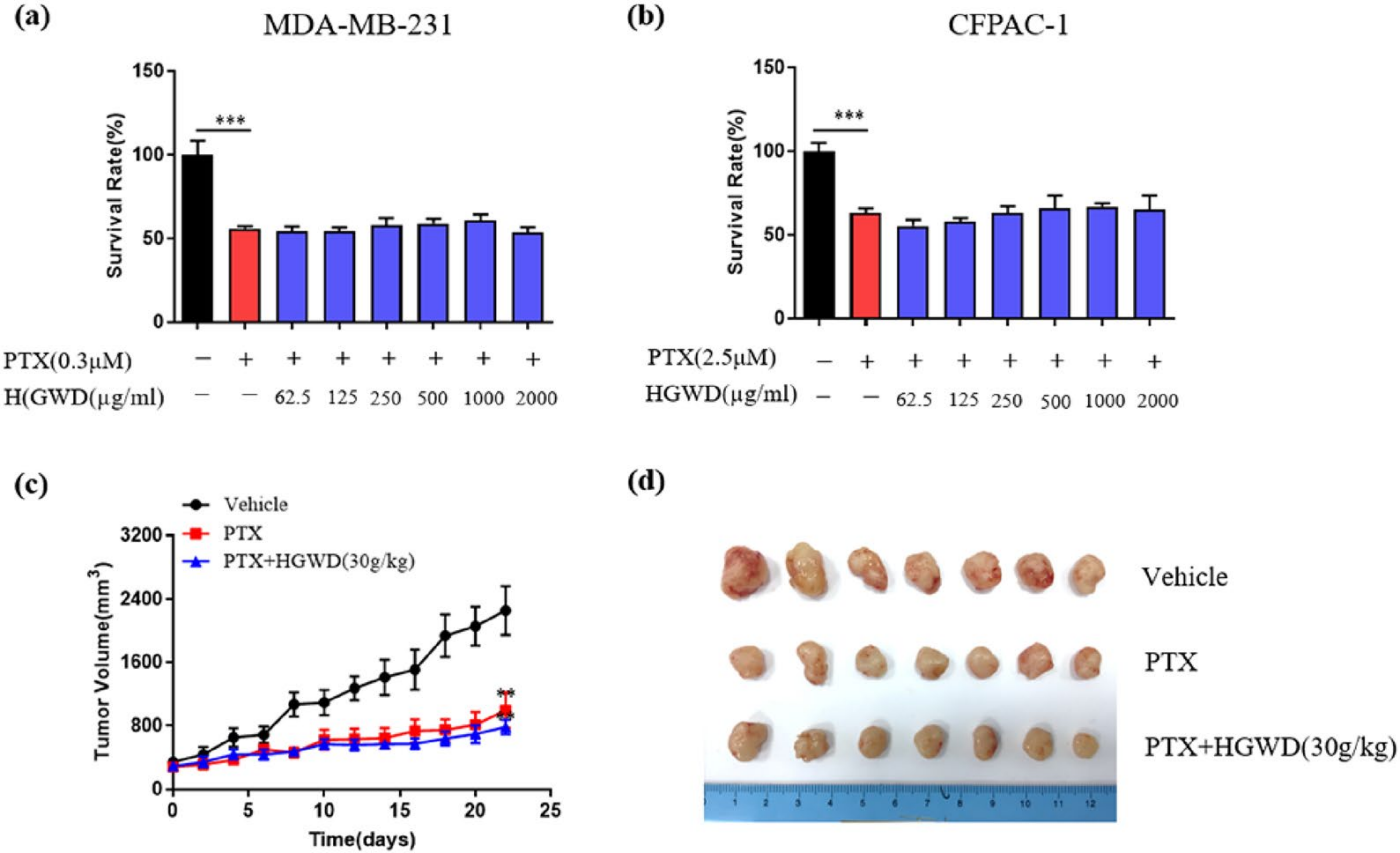
Evaluation of potential effects of HGWD on paclitaxel efficacy in tumor cell lines and a tumor growth model. **a–b** Tumor cell survival as measured by MTT assay in MDA-MB-231 breast cancer cells (**a**), and CFPAC-1 pancreatic cancer cells (**b**) (24 h). **c**–**d** Tumor size in nude BALB/c mice injected subcutaneously into the right flank with 4 × 10^6^ CFPAC-1 cells and treated with vehicle, paclitaxel (i.p., 10 mg/kg), paclitaxel associated with HGWD (i.g., 30 g/kg). ^**^P < 0.01, ^***^P < 0.001 vs. vehicle

After evaluating the neuroprotective effects of HGWD, we further investigated its effect on the chemotherapeutic activity of PTX. As shown in Fig. 10a and b, PTX suppressed the growth of MDA-MB-231 breast cancer cells and CFPAC-1 pancreatic cancer cells by 42.5 ± 3.3 and 36.8 ± 8.3%, respectively (*P* < 0.001). PTX-induced decreases in MDA-MB-231 and CFPAC-1 cell viability were significantly potentiated after co-incubation with HGWD (Fig. 10a, b). In a xenograft mouse model implanted with CFPAC-1 cells, tumor growth was significantly inhibited by PTX compared with that in the vehicle controls (*P* < 0.01) (Fig. 10c, d). Combinatorial use of PTX and HGWD led to little compromise on tumor growth inhibition of PTX. Both the in vitro and in vivo studies indicate that HGWD does not compromise the antitumor activity of PTX.

## Discussion

In this study, we revealed that HGWD significantly ameliorated PIPN and did not compromise the tumor cell-killing ability of PTX. To elucidate the underlying mechanism of HGWD against PIPN, network pharmacology was performed to analyse its potential active components and targets. The results of bioinformatics predictions and experimental validation showed that HGWD inhibited TLR4/NF-κB signalling and activated PI3K/Akt/Nrf2 pathways in PIPN rats. To our knowledge, this is the first study to elucidate the effect and protective mechanism of HGWD on PTX-induced neurotoxicity in vivo.

PIPN remains a difficult and unmet clinical problem. Herbal formula HGWD has been used to treat numbness, vibration sensation, cold sensation, and limb ache since the Eastern Han Dynasty, which is considered a potential complementary therapy providing neuronal protection in chemotherapy [17]. Recently, HGWD has been proven to be effective in treating oxaliplatin- and diabetic peripheral neuropathy in animal models and clinical studies [12, 13]. However, the effect of HGWD on PIPN with its pharmacological mechanism remains unclear, Thus, in this study, we tested whether HGWD alleviates PIPN and explore the underlying pharmacological mechanism.

A rat experimental model of PIPN was established and used because rats are physiologically closer to humans than mice [18]. HGWD effectively attenuated the mechanical allodynia and thermal hyperalgesia evoked by PTX (Fig. 2b, c). Moreover, pre-treatment with HGWD significantly rescued PTX-induced morphometric changes in peripheral sensory neurons, such as shrinkage of DRG nucleoli, axonal degeneration, and myelin damage in the sciatic nerve (Figs. 2c and 3). Furthermore, PTX damaged IENFs and led to IENF loss, which was primarily responsible for neuropathic pain [19]. As shown in Fig. 4a and b, HGWD remarkably attenuated this neurotoxic effect evidenced by increasing the density of PGP9.5. NGF is a well-known indicator for predicting the clinical severity and outcome of neuropathy in patients with CIPN. We found that HGWD treatment notably elevated serum NGF levels in PIPN rats. These results suggest that HGWD could effectively attenuates PIPN.

Increasing evidence has shown that inflammation is critical in the development of CIPN [20]. Excessive secretion of pro-inflammatory cytokines, including TNF-α, IL-1β, and IL-6, enhances PTX-induced peripheral sensitisation of sensory neurons and mechanical allodynia [21]. In this work, the modulation of HGWD on inflammatory cytokines was examined. Here, increases in IL-1β, IL-6, and TNF-α levels were observed in rat serum and DRG after PTX injection (Fig. 5). Interestingly, HGWD reversed the elevation of pro-inflammatory cytokines and increased IL-10, suggesting that its neuroprotection against PIPN is mediated, at least in part, by an anti-inflammatory activity (Fig. 5). In addition to inflammation, oxidative stress, which is caused by an imbalance between free radicals and antioxidant defences, has been identified to be closely associated with CIPN pathogenesis [22]. ROS scavengers, such as phenyl-N-tert-butylnitrone and acetyl-L-carnitine, have been shown to prevent and reverse PTX-induced pain [23]. In our experiment, SOD, MDA, and 8-isoprostane F2α levels were measured as markers of oxidative stress. As shown in Fig. 6**,** increased MDA and 8-isoprostane F2α levels and decreased SOD activity were observed in PIPN rodent models. PTX-induced elevation of oxidative stress was attenuated by HGWD. In addition, the PTX-induced increase in ROS generation in DRG was blocked by HGWD, suggesting that HGWD exerted antioxidant effect under PIPN conditions.

Next, network pharmacology was applied to elucidate the action mechanisms of HGWD. Finally, 30 active chemical ingredients with their physicochemical properties were screened from HGWD: 15 compounds from AR, 5 compounds from CR, 3 compounds from PRA, 11 compounds from JF, and 7 compounds from ZR (Table 1). Most of these active compounds have been reported to exert anti-inflammatory and antioxidant activities, indicating that these compounds may be key constituents for PIPN treatment. For instance, paeoniflorin exerts neuroprotective effect by attenuating oxidative stress through Nrf2 activation in diabetic peripheral neuropathy mice [24]. 6-Gingerol, cinnamic acid, and formononetin have been shown to play antioxidant and anti-inflammatory roles in several neurological diseases, such as ischemic brain damage, Alzheimer’s disease, and oxaliplatin-induced peripheral neuropathy [25–27]. Both KEGG pathway enrichment and network analyses predicted the PI3K-Akt and Toll-like receptor pathways as the key signalling pathways that mediate the protective effect of HGWD against PIPN (Fig. 7 and Additional file 1: Figure S2). To confirm the prediction, the effect of HGWD on the key proteins in these pathways was evaluated by western blotting. The results showed that HGWD significantly inhibited PTX-induced activation of the inflammatory signalling pathway TLR4/NF-κB and promoted the activation of PI3K/Akt-Nrf2 signalling in the DRG (Fig. 8).

TLR4 protein is a type of immune receptor expressed on cell surfaces and triggers innate immunity. Recently, TLR4 was shown to be involved in PTX-induced neuropathic pain. TLR4 deficiency or pharmacological blocking plays a neuroprotective role in PIPN [28, 29]. In our study, we showed that TLR4/NF-κB signalling pathway was activated in PIPN rats. HGWD treatment significantly inhibited TLR4 and MyD88 activation. Furthermore, HGWD decreased the expression of IKKα, and p-NF-κB (Fig. 9a). Thus, HGWD may play an important role in preventing PIPN by suppressing the activation of the TLR4/NF-κB signalling pathway. Nrf2 is a crucial regulator of defence against oxidative stress. Modulation of Nrf2 may alleviate oxidative stress injury in PIPN [30]. Notably, increasing evidence has suggested that PI3K/Akt is upstream of Nrf2 [31, 32]. Nrf2-mediated gene expression correlates with the activation of the PI3K/Akt pathway. As shown in Fig. 9b-c, our results suggest that HGWD attenuated PTX-induced oxidative stress partially by promoting PI3K/Akt-Nrf2 signalling.

Alongside the potential therapeutic effect of HGWD against PIPN, it is critical to determine whether HGWD interferes with the tumor cell-killing effect of chemotherapy. After confirming the neuroprotective action of HGWD against PIPN, we tested the effect of HGWD on the in vitro and in vivo antitumor efficacy of PTX. As expected, HGWD did not impair the antitumor activity of PTX in cancer cell lines and mice with transplanted xenografts (Fig. 10).

## Conclusion

**Fig. 11:**
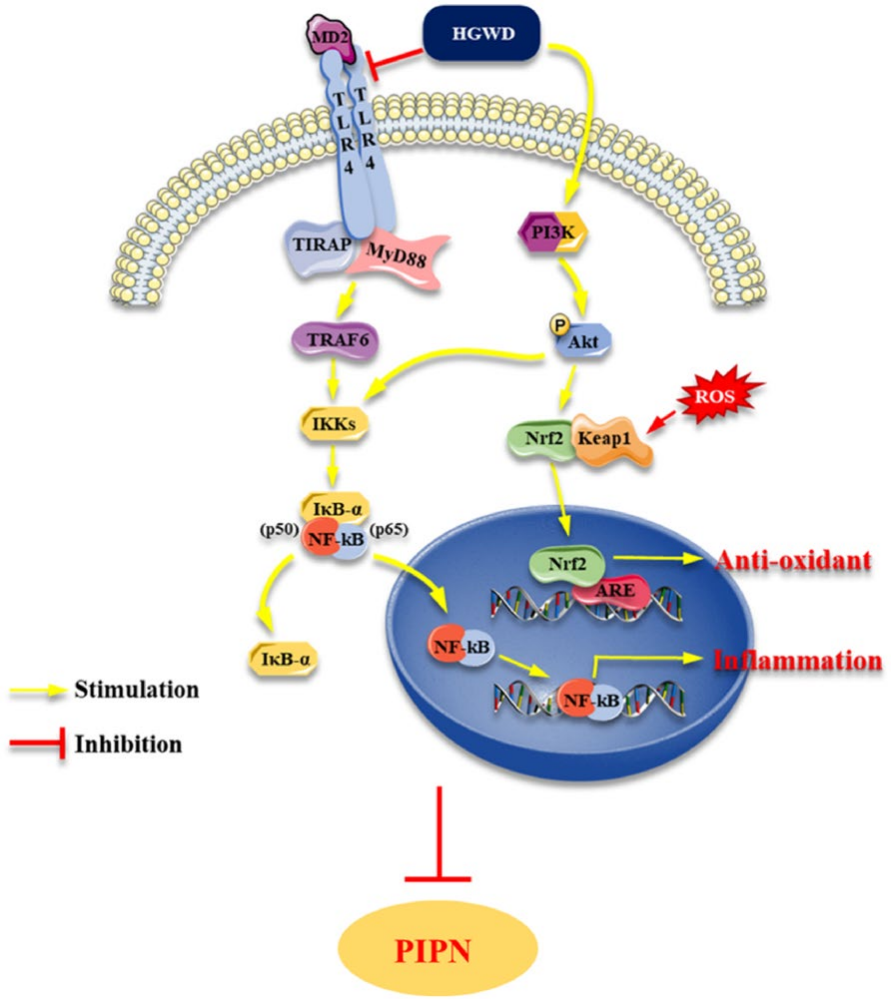
The connection between pathways and the key targets of HGWD acting on PIPN. Most of the targets have been validated

In summary, this study shows that HGWD, a representative prescription for treating “blood impediment”, can mitigate PTX-related neurotoxicity without compromising on antitumor activity of PTX. Network pharmacology combined with subsequent experimental validation suggested that HGWD exerted neuroprotective effect against PIPN partly by inhibiting inflammatory responses and activating antioxidant functions via regulating TLR4/NF-κB and PI3K/Akt-Nrf2 pathways (Fig. 11). This study provides a novel understanding of the classical herbal formula HGWD in the treatment of PIPN. More experiments are needed to study the in-depth mechanisms of HGWD on paclitaxel-related neurotoxicity in our future work.

## Supplementary Information

**Additional file 1**: **Table S1**. List of catalog numbers and assay ranges of ELISA kits. **Table S2**. Information of Public databases and its website involved in the study. **Table S3**. List of catalog numbers of antibodies in the western blotting test. **Table S4**. List of primers used for real-time PCR. **Figure S1**. Enrichment analysis for cell and tissue distribution of the potential common target proteins in PaGenBase. **Figure S2**. Results of target proteins of the HGWD formula with pathway in KEGG database.

## Data Availability

The datasets used in the current study are available from the corresponding author on reasonable request.

## Abbreviations

PIPN: Paclitaxel-induced peripheral neuropathy
HGWD: Huangqi Guizhi Wuwu decoction
CIPN: Chemotherapy-induced peripheral neuropathy
PTX: Paclitaxel
IENFs: Intraepidermal nerve fibres
TRP: Transient receptor potential channel
MAPK: Mitogen-activated protein kinase
ROS: Reactive oxygen species
AR: Astragali Radix
CR: Cinnamomi Ramulus
PRA: Paeonia Radix Alba
JF: Jujubae Fructus
ZR: Zingiberis Rhizoma
ADME: Absorption, distribution, metabolism, and excretion
OB: Oral bioavailability
C-T: Compound-target
T-P: Target-pathway
