# Supplementary material for: Herbal formula Huangqi Guizhi Wuwu decoction attenuates paclitaxel-related neurotoxicity via inhibition of inflammation and oxidative stress
Source: Chin Med. 2021 Aug 10;16:76. doi: 10.1186/s13020-021-00488-1 (PMC8353759; doi:10.1186/s13020-021-00488-1)
Supplement: Supplementary file 1 — Additional file 1: Table S1. List of catalog numbers and assay ranges of ELISA kits. Table S2. Information of Public databases and its website involved in the study. Table S3. List of catalog numbers of antibodies in the western blotting test. Table S4. List of primers used for real-time PCR. Figure S1. Enrichment analysis for cell and tissue distribution of the potential common target proteins in PaGenBase. Figure S2. Results of target proteins of the HGWD formula with pathway in KEGG database. [file 13020_2021_488_MOESM1_ESM.docx]

**Herbal Formula Huangqi Guizhi Wuwu** **Decoction** **Attenuates Paclitaxel-related** **Neurotoxicity via Inhibition of Inflammation and Oxidative Stress**

Zhangming Lv^1,2†^, Jiayun Shen^1,2†^, Xuejiao Gao^1,2^, Yonglan Ruan^1^, Jinying Ling^1^, Rongwei Sun^1^, Jingya Dai^1^, Haizhen Fan^1^, Xiaolan Cheng^1 *^, Peng Cao^1,2*^

^1^*Affiliated Hospital of Integrated Traditional Chinese and Western Medicine,* *Nanjing University of Chinese Medicine, Nanjing 210028, China*

^2^*College of Pharmacy, Nanjing University of Chinese Medicine, Nanjing 210029, China*

* Corresponding author. Tel./Fax: +86 25 85608666.

E-mail address: cao_peng@njucm.edu.cn (Peng Cao), chengxiaolan37@126.com (Xiaolan Cheng).

College of Pharmacy, Nanjing University of Chinese Medicine, Nanjing 210029, China

^†^These authors contributed to the work equally.

**Supplementary** **Tables**

**Table S1.** List of catalog numbers and assay ranges of ELISA kits.

| ELISA Kits | Catalog numbers | Assay ranges |
| --- | --- | --- |
| NGF | ERNGF | 20.58-15,000 pg/mL |
| 8-iso-PGF | ADI-900-010 | 6.1 pg/ml - 100000 pg/ml |
| SOD | S0101S | ＞0.5U/ml |
| MDA | S0131S | 1-200 μM |
| IL-1β | 70-EK301BHS-96 | 7.81-500 pg/ml |
| IL-6 | 70-EK306HS-96 | 7.81-500 pg/ml |
| TNF-α | 70-EK382HS-96 | 7.81-500 pg/ml |
| IL-10 | 70-EK310HS-96 | 6.25-400 pg/ml |

**Table S2.** Information of Public databases and its website involved in the study.

| Databases | Website Address |
| --- | --- |
| TCMSP | http://lsp.nwu.edu.cn/tcmspsearch.php |
| TCMID | http://119.3.41.228:8000/tcmid/ |
| TargetNet | http://targetnet.scbdd.com |
| DrugBank | https://go.drugbank.com/ |
| UniProt | http://www.uniprot.org |
| OMIM | https://omim.org/ |
| GeneCards | https://www.genecards.org/ |
| NCBI-gene | https://www.ncbi.nlm.nih.gov/gene |
| DisGENET | https://www.disgenet.org/ |
| KOBAS | http://kobas.cbi.pku.edu.cn/kobas3 |
| Metascape | <https://metascape.org> |
| String | https://string-db.org/ |

**Table S3.** List of catalog numbers of antibodies in the western blotting test.

| Antibodies | Catalog numbers |
| --- | --- |
| Anti-TLR4 | ab13867 |
| Anti-MyD88 | ab2064 |
| Anti-IKK alpha | ab227852 |
| Anti-NF-kB p65 | ab239882 |
| Anti-Phospho-NF-κB p65 | AN371 |
| Anti-PI3K | ab191606 |
| Anti-Akt | ab38449 |
| Anti-Nrf2 | ab137550 |
| Anti-Keap1 | ab139729 |
| Anti-Heme Oxygenase 1 | ab223349 |
| Anti-Tubulin | ab56676 |
| Goat Anti-Mouse IgG H&L (HRP) | ab205719 |
| Goat Anti-Rabbit IgG H&L (HRP) | ab205718 |

**Table S4.** List of primers used for real-time PCR.

| Gene names | Primers |
| --- | --- |
| IL-1β | Sense:5’-CCTCTGCCAAGTCAGGTCTC-3’ |
|  | Antisense:5’-GAATGTGCCACGGTTTTCTT-3’ |
| IL-6 | Sense:5’-CACAAGTCCGGAGAGGAGAC-3’ |
|  | Antisense:5’-CAGAATTGCCATTGCACAAC-3’ |
| IL-10 | Sense:5’-CTGCTCTTACTGGCTGGAGTGAAG-3’ |
|  | Antisense:5’-TGGGTCTGGCTGACTGGGAAG-3’ |
| TNF-α | Sense:5’-CTTCTGTCTACTGAACTTC-3’ |
|  | Antisense:5’-AAGATGATCTGAGTGTGA-3’ |
| β-actin | Sense:5’-ATCGCTGACAGGATGCAGAA-3’ |
|  | Antisense:5’-TAGAGCCACCAATCCACACAG-3’ |

**Supplementary** **Figures**

**
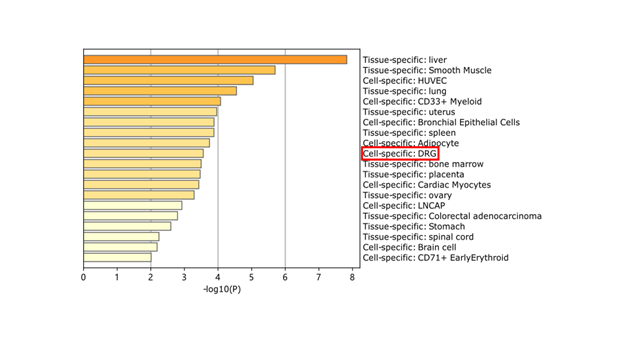
**

**Figure S1.** Enrichment analysis for cell and tissue distribution of the potential common target proteins in PaGenBase. Enrichment analysis of 158 potential common target proteins identified in the PaGenBase was conducted on Metascape website. All genes in the genome have been used as the enrichment background. Terms with a p-value < 0.01, a minimum count of 3, and an enrichment factor > 1.5 are collected and grouped into clusters based on their membership similarities.

**
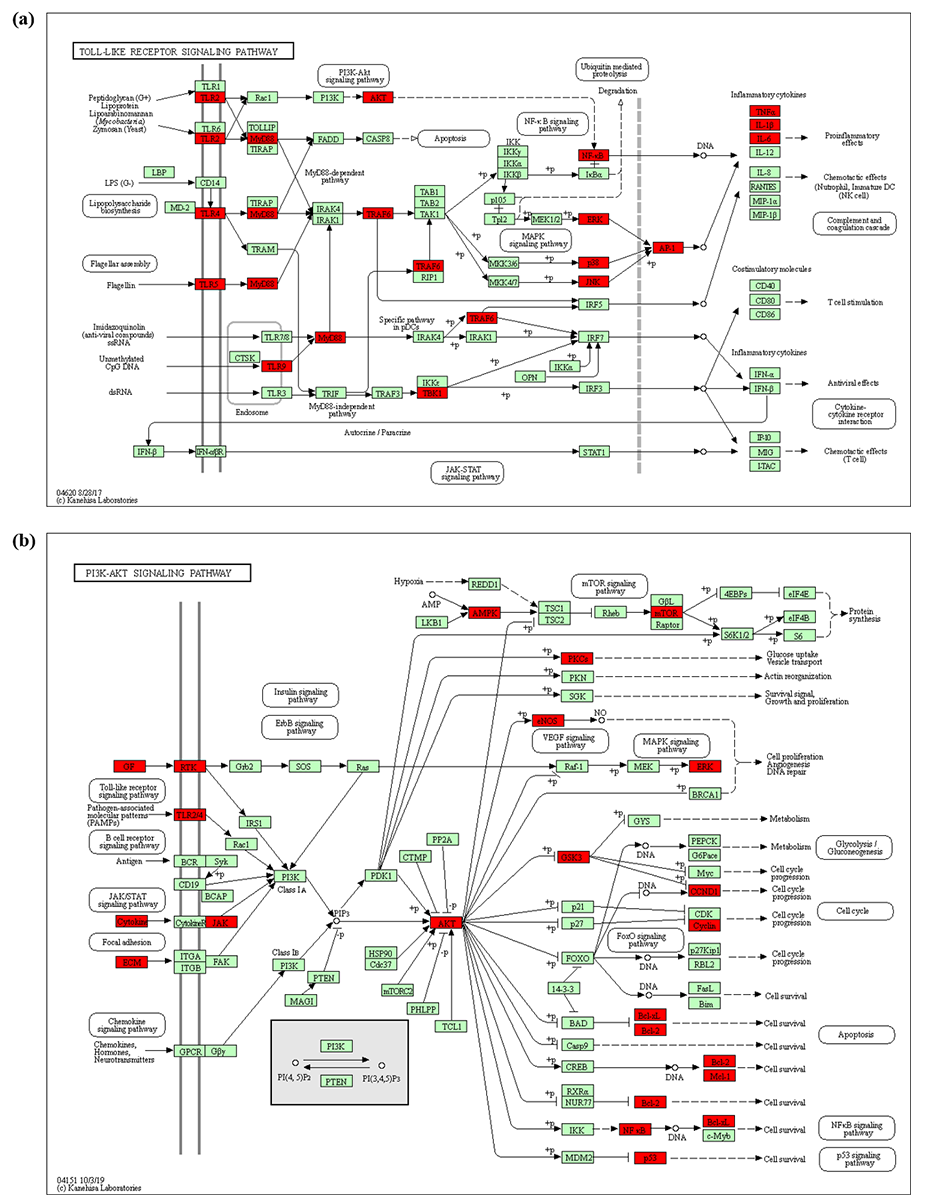
**

**Figure S2**. Results of target proteins of the HGWD formula with pathway in KEGG database. (a) Targets and frequency of HGWD active ingredients in Toll-like receptor signaling pathway. (b) Targets and frequency of HGWD active ingredients in PI3K-AKT signaling pathway.
